# A database of nutritional strategies of nanoplankton genera present in North American lake surface waters

**DOI:** 10.1093/plankt/fbae035

**Published:** 2024-07-10

**Authors:** Philippe Le Noac’h, Beatrix E Beisner

**Affiliations:** Department of Biological Sciences and Interuniversity Research Group in Limnology/Groupe de Recherche Interuniversitaire en Limnologie (GRIL), University of Quebec at Montreal, Montreal, C.P. 8888, Succ. Centre-Ville, Montréal, QC, H3C 3P8, Canada; Department of Biological Sciences and Interuniversity Research Group in Limnology/Groupe de Recherche Interuniversitaire en Limnologie (GRIL), University of Quebec at Montreal, Montreal, C.P. 8888, Succ. Centre-Ville, Montréal, QC, H3C 3P8, Canada

**Keywords:** nanoplankton, North American lakes, nutrition strategies, phago-mixotrophy

## Abstract

A database of nutritional strategies of nanoplankton genera present in North American lake surface waters is presented. This work represents an integrated and updated database of nutritional strategies for nanoplankton genera commonly found in surface waters of North American lakes. We tabulate the nutritional strategies (autotroph, phago-mixotroph and phago-heterotroph) for nanoplankton genera identified during several pan-continental lake surveys: the EPA-NLA surveys conducted in 2012 and 2017 across the continental USA and the NSERC Canadian Lake Pulse survey campaign conducted from 2017 to 2019. We expect that this work will serve others in the plankton community interested in assessing nanoplankton feeding strategies.

## INTRODUCTION

Constitutive mixotrophy, the ability for organisms to acquire their resources through both photoautotrophic pathways and prey ingestion, is a widespread functional trait within nanophytoplankton communities. It is found across all aquatic environments (Flynn *et al.*, 2013; Faure *et al.*, 2019) and is important to consider in assessments of critical ecosystem processes like primary production or carbon fluxes (Mitra *et al.*, 2014). While the study of nanophytoplankton mixotrophy has generated increased levels of interest over the last two decades, including this special issue, its place within freshwaters is still not well understood, mainly because of a lack of information on the nutritional strategies of nanophytoplankton taxa. In freshwaters, nanophytoplankton are generally defined as autotrophic plankton between 2 and 20 μm, although in the case of some colonial species or diatoms, overall maximum linear dimensions can range larger, up to 100 μm. In recent years, several nanoplankton functional trait databases have been published: Laplace-Treyture *et al.* (2021), Rimet and Druart (2018), HELCOM (Olenina *et al.*, 2006) and Mitra *et al.* (2023). However, such databases are sometimes inconsistent with each another, do not necessarily focus on the nanoplankton size-fraction of mixoplankton organisms, nor include taxa commonly found in freshwaters (primarily lakes), as they are geographically specific or marine.

Large-scale sampling campaigns across North America have generated community compositional data for hundreds of lakes and reservoirs across large environmental gradients. We recently analysed data from these surveys, focusing on a comparison of mixotrophic nanophytoplankton across the many ecoregions and environmental drivers; the largest-scale assessment of the prevalence of phago-mixotrophy in lakes to date (Le Noac’h *et al.*, 2024). Overall, we found that nutrient availability was most strongly related to phago-mixotrophy in North American lake surface waters. Here our objective is to present the nutritional strategy database used to characterize the nanoplankton communities in that continental study. We thus assemble for the first time, a database on (photo-phago-)mixotroph and photo-autotrophic nanophytoplankton for the North American continent. Moreover, we have found sources for the nutritional strategy attributions where classification was doubtful in previous databases.

Given that most mixoplankton research has focused on marine ecosystems (e.g. Flynn *et al.* 2019), we also hope that this database will aid others in furthering mixoplankton research in freshwaters at both more localized studies as well as across broad continental or global scales. Millette *et al.* (2023) outlined five main priorities for mixoplankton research, geared toward improve understanding of: mixoplankton evolution, traits and trade-offs in mixotrophic metabolism, biogeography of mixoplankton, biogeochemical consequences of mixotrophy and improving identification of phago-mixotrophic activity. By summarizing current knowledge on phago-mixotrophy on a large number of taxa common to North American lakes and beyond, this database will serve to advance these priorities, given that such broad-scale taxonomic-trait information assembled in a single place is lacking.

## METHODS

### Survey methods

Nanophytoplankton included in this study were those censused by three large-scale lake surveys: the US Environmental Protection Agency (EPA)-National Lake Assessment conducted in 2012 and 2017 across the USA (EPA NLA, 2009) and the NERSC-Lake Pulse (LakePulse) survey in Canada from 2017 to 2019 (Huot *et al.*, 2019). Both surveys measured many biotic and abiotic variables, including identification of nanophytoplankton communities using the same sampling methodology. Nanophytoplankton community composition at each site was assessed from integrated tube water samples taken over the first 2 m of the water column or down to the deepest limit of the photic zone (whichever was smaller). Nanophytoplankton were identified by taxonomists and enumerated using the Ütermohl method (Utermöhl, 1931) on an inverted microscope (up to 1000× magnification). Sampling and identification procedures for the nanoplankton in all surveys focused primarily on photosynthetic taxa. Consequently, the present nutrition database features relatively few purely heterotrophic taxa relative to autotrophic and phago-mixotrophic taxa. From the list of all nanoplankton identified across all surveys, we retained all unique nanoplankton genera and inferred their nutritional strategies using a combination of taxonomic inference, queries on preexisting trait databases and literature review.

### Classification methods

Given that species-level information is often lacking or inaccurate in both count data and in assessments of mixoplankton, we focus our database classification at the genus level. Cyanobacteria and diatoms were always classified as (photo)autotrophic as they lack the physiological and cellular structures to ingest prey. It should be noted that these groups are likely carrying out osmotrophy as well as autotrophy, but the information available by taxon on this capacity is too preliminary to allow for a confirmed assignment; thus they are referred to simply as autotrophs in the database. Thus, our database considers exclusively photo-phago-mixotrophs (and not photo-osmo-mixotrophs) where mixotrophy is confirmed. Nutritional strategies for other groups were deduced using several methods. A preliminary search was conducted using three published nanoplankton functional trait databases with good coverage of freshwater taxa: Laplace-Treyture *et al.* (2021), Rimet and Druart (2018) and HELCOM (Olenina *et al.*, 2006). Laplace-Treyture *et al.* (2021) compiled functional trait values for 639 freshwater taxa in French waterbodies, including a nutrition trait with three categories: *Autotroph*, *Mixotroph* and *Heterotroph*. Rimet and Druart (2018) compiled these same trait categories for >1200 phytoplankton taxa found in European, mainly alpine and high-altitude temperate lakes and waterbodies. The HELCOM consortium database (Olenina *et al.*, 2006) primarily focuses on marine taxa. For a given genus from our surveys, if at least two of the existing trait databases reported the same nutritional strategy, this strategy was accepted.

When a genus was not found in any of these existing databases or was present in only one, we attempted to infer its nutritional strategy from those of closely related taxa from the same taxonomic family (preferably) or order. For the nutritional strategy to be inferred this way, the initial database search had to have yielded coherent reports of strategy for at least two taxa from the same family (or order) and the nutritional strategies of all related taxa identified had to match. Our accompanying database identifies the taxonomic level for our inferences (Appendix 1).

When the databases collectively reported conflicting information on a genus’ strategy and when the taxonomic inference did not yield any results, a literature search was conducted. Results from ingestion experiments were prioritized. We were careful to distinguish between reports of osmo-mixotrophy from phago-mixotrophy, the latter of which was the focus of Le Noac’h *et al.* (2024) and this study. If no information was found for a genus, its nutritional strategy was inferred from those of closely related taxa from the same family or order for which we could find literature information. Pigmented genera not included in any of the three databases, for which we could not find publications describing their feeding behavior, and that had no taxonomically close relative with an identified nutrition strategy, were classified as photo-autotrophs. For each genus, the reasoning for nutrition strategy attribution after the literature search, including relevant references, is reported in the final database (Appendix 1). The last date of data verification and archive update were done on 15 April 2024.

## RESULTS AND DISCUSSION

We present an integrated and updated database of nutritional strategies for nanoplankton genera commonly found in surface waters of North American lakes. This database ascribes nutritional strategies to nanoplankton genera identified during three pan-continental lake surveys. Genera were classified as either (photo)autotroph, potential constitutive phago-mixotroph or, in a few instances, as phago-heterotroph (i.e. non-pigmented taxa that acquire their carbon solely through phagotrophy). Future research should work toward greater identification of mixoplankton at the species level.

**Fig. 1:**
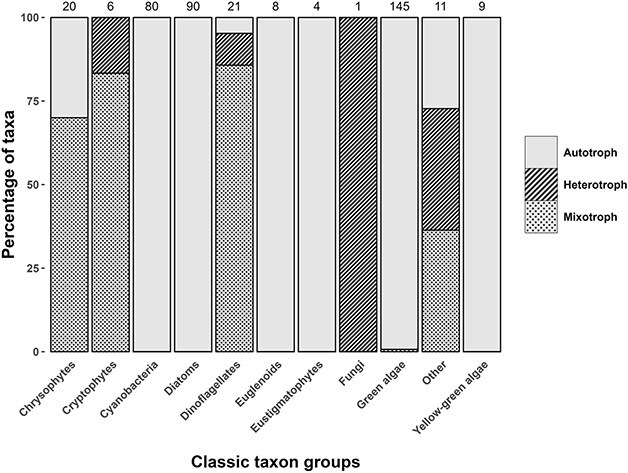
Trophic classification of the taxa in our database based on our updated literature review showing the proportion of autotrophic, heterotrophic or mixotrophic assignments by taxa using their classical names (newer names listed in the database). Numbers above each column indicate the number of genera associated with each group.

This database provides this nutritional strategy information for close to 400 genera present in the surface waters of North American lakes and reservoirs and complements our recent continental-scale assessment of the prevalence of mixotrophy in lake nanophytoplankton communities, spanning hundreds of sites in Le Noac’h *et al.* (2024). The majority of mixotrophy was observed in the taxa classically called: Dinoflagellates, Cryptophytes and Chrysophytes with some representation in the Chlorophytes and the “Other” group (based on classic group names; but see the database for more recent classifications) (Fig. 1). This “Other” group was composed of Haptophytes, Ochrophytes and Choanozoa.

Our goal in publishing the database is to aid other researchers to expand work on mixoplankton in freshwater ecosystems across North America and globally. We hope others will use this work for future studies in freshwater nanophytoplankton communities. The database should serve to help better characterize functional relationships, food web fluxes and community assembly and diversity in nanoplankton communities under different environmental conditions or food web configurations; and these at both local and biogeographical scales.

## Supplementary Material

Appendix_1_text_clean_fbae035

NanoplanktonNutritionStrategiesDBLeNoachBeisner2024_fbae035

NanoplanktonNutritionStrategiesDB_V2_fbae035

ReadmeCSV_metadata_LeNoachBeisner_fbae035

## Data Availability

All data are incorporated into the article and its online supplementary material.
